# Numerical Iterative Design and Analysis of Double Freeform Surface Telecentric F-Theta Lens

**DOI:** 10.3390/s25164949

**Published:** 2025-08-10

**Authors:** Li Wang, Ke Chen, Xueliang Kang, Hongmei Zheng

**Affiliations:** 1 School of Mechanical Engineering, Hefei University of Technology, Hefei 230009, China; lilyxyz@163.com (L.W.); hongmei891121@163.com (H.Z.); 2 College of Mechanical Engineering, North Minzu University, Yinchuan 750021, China; 3 College of Electrical and Information Engineering, North Minzu University, Yinchuan 750021, China; kxl@mail.ustc.edu.cn

**Keywords:** laser scanning system, f-theta lens, double freeform surfaces lens, numerical iterative design

## Abstract

**Highlights:**

Based on the ray mapping relationship of a telecentric f-theta lens, a numerical iterative design method of a double freeform surface f-theta lens is proposed in this paper. When the specific design indexes and the appropriate iterative starting data are given, the two freeform surfaces of this f-theta lens can be obtained directly and simultaneously by employing this design method. The simulation analysis demonstrates that the designed f-theta lens has a controllable distortion and high transmittance. This numerical iterative method provides a different idea for the design of an f-theta lens and can greatly simplify the design process.

**What are the main findings?**

- A numerical iterative design method of a double freeform surface telecentric f-theta lens is proposed.
- The two freeform surfaces of this f-theta lens can be obtained directly and simultaneously.

**What is the implication of the main finding?**

- The designed f-theta lens has a controllable distortion and high transmittance.
- It provides a new design concept for lens design.

**Abstract:**

As one of the most important optical components of a laser scanning system, the f-theta lens group largely determines the working range and scanning accuracy of this system. In this paper, a numerical iterative design method of a double freeform surface telecentric f-theta lens is proposed. Then the scanning linearity and optical transmittance of the designed f-theta lens are simulated and analyzed. The results show that the f-theta lens designed by this method has a compact structure, large scanning area, extremely high optical transmittance over the entire field of view, and stable, controllable error of scanning linearity. Different from the optimization design method of the traditional f-theta lens group, the two freeform surfaces of this f-theta lens can be obtained directly and simultaneously by using this numerical iterative method, which consumes less computational resources and significantly reduces the design complexity.

## 1. Introduction

The *f*-*θ* lens group is a key component of laser scanning systems and is widely used in fields such as laser measurement [1], laser detection [2], and laser printing [3]. The function of the *f*-*θ* lens group is to transform the uniform rotation of the laser beam reflected by the rotating polygonal mirror, into the linear displacement of image spot in the working plane, so as to achieve high-precision scanning or imaging. The *f*-*θ* lens group has a decisive impact on the performance of the laser scanning system. Its angle of field and focal length determine the working range, while its optical transformation linearity determines the scanning accuracy of the laser scanning system. The field curvature and transmittance of the *f*-*θ* lens group are also important, because they can significantly affect the image quality in the working plane of the system. Therefore, how to optimize the structure and improve the optical performance of the *f*-*θ* lens group is one of the current hotspots in the research field of laser scanning.

The conventional *f*-*θ* lens group is usually composed of several spherical convex and concave lenses according to certain optical principles [1]. This structure can effectively minimize aberrations, such as distortion. Thus, a lot of research studies have focused on the structure design and performance optimization of this kind of *f*-*θ* lens group. And many corresponding high-performance *f*-*θ* lens groups have been designed for various application requirements [4,5,6,7,8,9]. However, these conventional *f*-*θ* lens groups with multi-lens structures have defects such as large size, complex assembly, low transmittance, and low design freedom.

Other studies have shown that the introduction of aspheric optical components into the design of an *f*-*θ* lens group can effectively overcome the aforementioned defects [10,11,12]. Especially in recent years, with the development of freeform surface machining and measurement techniques [13,14,15,16,17,18], freeform surface optical components have been widely used in the optical design of various imaging systems and non-imaging systems [19,20,21,22]. The *f*-*θ* lens group with freeform surfaces can further correct special aberrations and significantly improve resolution. These systems also have a more compact structure and larger scanning range [23,24]. Even a single lens can replace the traditional *f*-*θ* multi-lens group and realize the linear optical transformation function, of which the front and back surfaces are both freeform surfaces [25,26,27,28,29]. Among all kinds of design methods of freeform surfaces, the point-by-point design method is the most direct and efficient [29,30,31,32,33]. Its key idea is to calculate surface points and normal vectors using the Snell law and the tangent continuity constraints. The method are well-established and widely applied in *f*-*θ* lens and light distribution elements. But most of them have only one freeform surface. Even for those double freeform surface lens, the two freeform surfaces are designed successively and independently by employing this method [29]. In other words, this point-by-point design method established in prior studies is only applicable to obtaining one freeform surface at a time.

In this paper, combined with the design theory of a telecentric *f*-*θ* lens and double freeform surface lens, a numerical iterative design method of a double freeform surface telecentric *f*-*θ* lens is proposed, and the structure design and performance analysis of this *f*-*θ* single lens is completed. Especially, the two freeform surfaces’ contours of the designed *f*-*θ* lens are obtained directly and simultaneously in the design process. The numerical iterative design method of a double freeform surface *f*-*θ* lens is described in Section 2. The optical performance of the designed *f*-*θ* lens is simulated and analyzed in Section 3. And the research conclusion of this paper is given in Section 4.

## 2. Design Methodology

**Figure 1:**
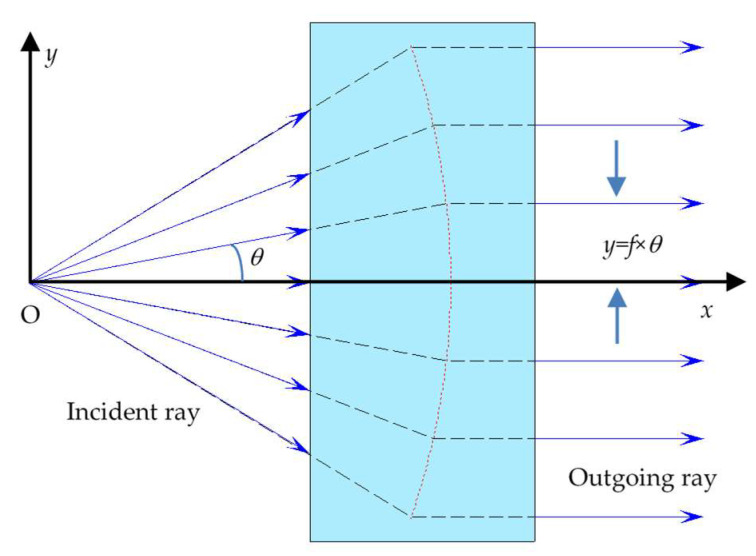
Mapping relationship between incident and outgoing rays in the *f*-*θ* transformation system.

As shown in Figure 1, in an *f*-*θ* optical transformation system, the ray is emitted from the focus point O with a scanning angle, i.e., an inclination angle *θ*. After being transformed by the system, the corresponding outgoing ray must be parallel to the optical axis of the system. The distance between the outgoing ray and the optical axis, i.e., the height of the outgoing ray, is given by the equation *y* = *f* × *θ*, where *f* represents the focal length of the *f*-*θ* optical transformation system.

The red dotted curve is formed by the intersections of the extensions of the incident rays and the reverse extensions of the outgoing rays in the *f*-*θ* transformation system in Figure 1. If the *f*-*θ* transformation system is rotationally symmetrical, this curve represents a surface that is rotationally symmetrical with respect to the optical axis, which is the object principal surface (it should be noted that it is not a plane) of the *f*-*θ* transformation system.

In geometrical optics, the change in the direction of the ray always occurs on a reflective or refractive surface. Thus, if the *f*-*θ* transformation system has only one optical surface, this optical surface is most likely to be the surface shown by the red dotted curve in Figure 1. But it is clear that on this surface, the relationship between the incident and outgoing rays is neither in accordance with the laws of reflection nor refraction. It follows that an *f*-*θ* transformation system should have at least two optical surfaces.

Based on the discussion above, if the *f*-*θ* transformation system has only two optical surfaces, they must be located on either side of the surface shown by the red dotted curve in Figure 1 and be refractive surfaces. In other words, such an *f*-*θ* transformation system must be a lens.

The following will describe the process of solving for the contours of the front and back surfaces of the *f*-*θ* lens.

**Figure 2:**
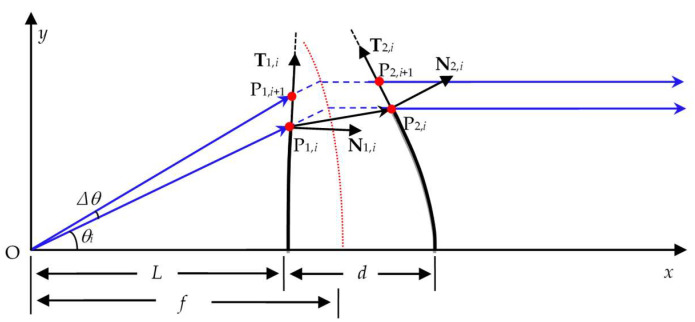
Iterative design of the contours of the double freeform surface *f*-*θ* lens.

As shown in Figure 2, take the point where the light source is located as the coordinate origin O which corresponds to the incident point of the laser on the surface of the rotating polygonal mirror.

As a matter of fact, the spatial position of the incident point on the polygonal mirror exhibits angular dependence due to the offset between the mirror surface and the rotational axis of the polygon in a laser scanning system. Consequently, the center principal rays with different scanning angles deviate from passing the fixed point O. And this deviation is asymmetrical between the center principal rays with positive and negative scanning angles. This geometric asymmetry will introduce inevitable differences in the contour design of the *f*-*θ* lens between the +*y* and −*y* regions. Thus, the size of the entrance pupil aperture must be considered in the *f*-*θ* lens design. Fortunately, the entrance pupil can be regarded as a point when the field of view of the *f*-*θ* lens or the polygonal mirror is small enough. Although the center principal rays with different scanning angles emit from different incident points and cross with each other in the near area, these rays will separate after propagating a distance and be incident on the front surface of the *f*-*θ* lens sequentially in accordance with the scanning angles. Therefore, even for a wide-view *f*-*θ* lens or a large polygonal mirror, the method described below is still effective for designing the surface contour of an *f*-*θ* lens with double freeform surfaces.

The range of scanning angles of the rays is the field of view of the *f*-*θ* lens to be designed and bisected by the *x*-axis. According to the symmetry, the *x*-axis is also the optical axis of the *f*-*θ* lens. It is assumed that this *f*-*θ* lens is placed in air and has a refractive index *n*.

The distance from the coordinate origin to the center of the front surface of the *f*-*θ* lens is the object distance *L*, and the distance between the centers of the front and back surfaces of the lens is the center thickness *d* of the lens. Then the coordinates of the center point P_1,0_(*x*_1,0_, *y*_1,0_) of the front surface and the center point P_2,0_(*x*_2,0_, *y*_2,0_) of the back surface of the lens are, respectively,

(1) $$\left\{\begin{array}{l} \left(x_{1,0},y_{1,0}\right)=(L,0),\\ \left(x_{2,0},y_{2,0}\right)=(L+d,0). \end{array}\right.$$

The unit tangential vector **T**_1,0_ of the front surface at point P_1,0_ and the unit tangential vector **T**_2,0_ of the back surface at point P_2,0_ are both perpendicular to the *x*-axis. Therefore, they are

(2) $$T_{1,0}=(0,1)=T_{2,0}=(0,1).$$

These coordinates and vectors are the starting data for the iterative algorithm.

In order to ensure that the iterative algorithm is convergent, it can be clearly seen from Figure 2 that the front and back surfaces of the *f*-*θ* lens must be located on either side of the object principal surface. In other words, the object distance *L* must be less than the focal length *f* of the lens, and the thickness *d* of the lens must be limited by *L + d > f*. Of course, this is only a necessary condition for the convergence of the iterative algorithm, but not a sufficient condition. With the increase in the scanning angle, both the front and back surfaces will bend closer to the object principal surface. Therefore, only if the iterative starting data *L* and *d* are set appropriately, would the front and back surfaces of the *f*-*θ* lens be located on either side of the object principal surface over the entire field of view. And finally, a processable lens would be generated.

The inclination angle (i.e., the scanning angle) of the *i*th incident ray emitted from the origin is denoted as *θ_i_*. Thus the unit vector of this ray is

(3) $$A_{1,i}=(\cos\theta_{i},\sin\theta_{i})$$

where the first subscript 1 indicates that the ray is before the front surface of the *f*-*θ* lens, and the second subscript *i* indicates that the ray is the *i*th one. Since this lens is placed in air, this vector is also the incident vector.

It is assumed that the two incident points P_1,*i*_(*x*_1,*I*_, *y*_1,*i*_) and P_2,*i*_(*x*_2,*I*_, *y*_2,*i*_) corresponding to the *i*th ray on the front and back surfaces of the *f*-*θ* lens are known, where the first subscript 1 indicates that the point is on the front surface, and 2 indicates that the point is on the back surface of the *f*-*θ* lens. The line segment connecting these two points is the path of the refracted ray inside the lens, and its unit vector is

(4) $$A_{12,i}=\left(x_{2,i}-x_{1,i},y_{2,i}-y_{1,i}\right)/\left|\left(x_{2,i}-x_{1,i},y_{2,i}-y_{1,i}\right)\right|$$

where the first subscript 12 indicates that the ray is between the front and back surfaces of the *f*-*θ* lens. Considering the refractive index *n* of the *f*-*θ* lens, the refraction vector corresponding to this refracted ray is denoted as

(5) $${A^{\prime }}_{12,i}=nA_{12,i}=n\left(x_{2,i}-x_{1,i},y_{2,i}-y_{1,i}\right)/\left|\left(x_{2,i}-x_{1,i},y_{2,i}-y_{1,i}\right)\right|$$

The description above shows that for the refraction at point P_1,*i*_ on the front surface of the *f*-*θ* lens, both the incident vector **A**_1,*i*_ and the refraction vector **A**′_12,*i*_ have been determined. Therefore, the normal direction of the front surface at this point is inevitably required.

According to the refraction law in vector form, the unit normal vector of the front surface at point P_1,*i*_ is

(6) $$N_{1,i}=\left({A^{\prime }}_{12,i}-A_{1,i}\right)/\left|{A^{\prime }}_{12,i}-A_{1,i}\right|$$

The unit tangential vector **T**_1,*i*_ of the front surface at point P_1,*i*_ is perpendicular to the unit normal vector **N**_1,*i*_. Thus, there is

(7) $$\left\{\begin{array}{l} T_{1,i}\cdot N_{1,i}=0,\\ \left|T_{1,i}\right|=1. \end{array}\right.$$

Combining the above equations from (3) to (7), the unit tangential vector **T**_1,*i*_ at that point P_1,*i*_ on the front surface of the lens can be solved.

As can be seen from Figure 2, if the angular discrete interval ∆*θ* is small enough, the intersection of the *i* + 1th incident ray with the inclination angle of *θ_i_*_+1_ = *θ_i_* + ∆*θ* and the tangent line of the front surface at point P_1,*i*_ can be regarded as the *i* + 1th point P_1,*i*+1_(*x*_1,*i*+1_, *y*_1,*i*+1_) on the front surface of the designed *f*-*θ* lens. On one hand, the point P_1,*i*+1_ locates on the incident ray, so there is

(8) $$y_{1,i+1}/x_{1,i+1}=\tan\theta_{i+1}$$

On the other hand, the point P_1,*i*+1_ locates on the tangent line of the front surface at point P_1,*i*_, and its coordinates must satisfy

(9) $$\left(x_{1,i+1}-x_{1,i},y_{1,i+1}-y_{1,i}\right)/\left|\left(x_{1,i+1}-x_{1,i},y_{1,i+1}-y_{1,i}\right)\right|=T_{1,i}$$

According to Equations (8) and (9), the coordinates of the *i* + 1th point P_1,*i*+1_ on the front surface of the designed *f*-*θ* lens can be solved.

The above procedure shows how to determine the *i* + 1th point by numerical iteration according to the *i*th point on the front surface of the *f*-*θ* lens.

The procedure of determining the *i* + 1th point on the back surface of the *f*-*θ* lens is similar and described briefly as follows.

The incident ray at point P_2,*i*_ on the back surface of the *f*-*θ* lens is also the refracted ray at point P_1,*i*_ on the front surface. All the outgoing rays on the back surface of the telecentric *f*-*θ* lens are parallel to the optical axis, so the unit vector of the outgoing ray at point P_2,*i*_ is

(10) $$A_{2,i}=(1,0)$$

This vector is also the refraction vector at point P_2,*i*_ on the back surface.

Thus, for the refraction at point P_2,*i*_ on the back surface of the *f*-*θ* lens, both the incident vector **A**′_12,*i*_ and the refraction vector **A**_2,*i*_ are determined. Similarly, applying the refraction law in vector form, the unit normal vector **N**_2,*i*_ and the unit tangential vector **T**_2,*i*_ of the back surface at point P_2,*i*_, as well as the *i* + 1th point P_2,*i*+1_(*x*_2,*i*+1_, *y*_2,*i*+1_) on the back surface, can be solved sequentially.

According to the above ideas, the coordinates of the *i* + 1th pair points P_1,*i*+1_ and P_2,*i*+1_ can be iteratively calculated from the coordinates of the *i*th pair points P_1,*i*_ and P_2,*i*_ on the front and back surfaces of the *f*-*θ* lens, until the inclination angle *θ_i_*_+1_ of the *i* + 1th incident ray exceeds the half-angle of field *θ*_max_ of the designed *f*-*θ* lens.

**Figure 3:**
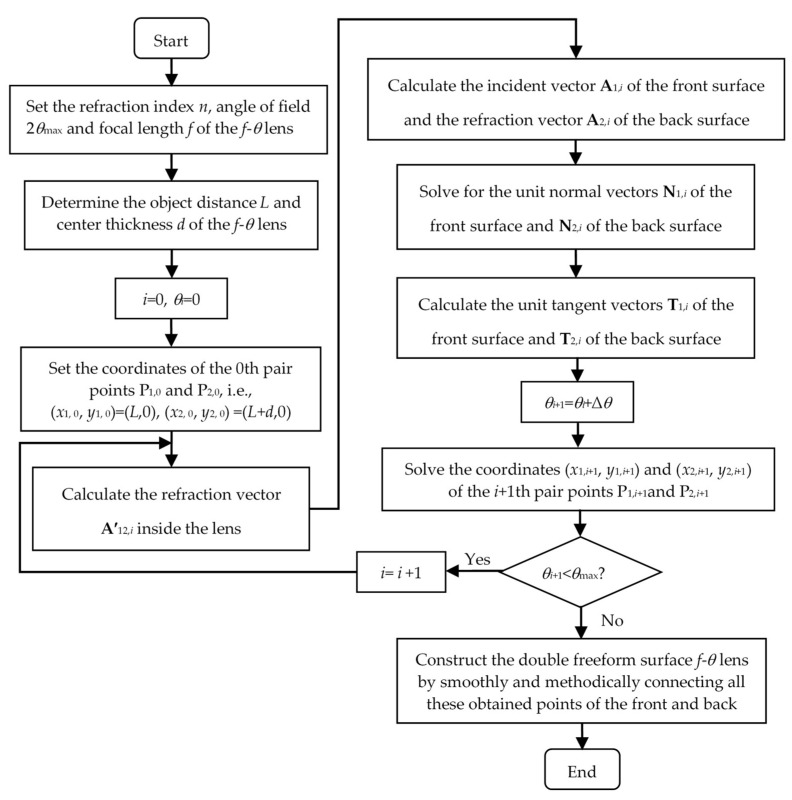
Iterative design flow of the double freeform surface *f*-*θ* lens.

Because the front and back surfaces of this designed *f*-*θ* lens are both freeform surfaces, the lens is called a double freeform surface *f*-*θ* lens. The iterative design flow is shown in Figure 3. As long as the coordinates of the 0th pair points of the front and back surfaces, that is, the centers of the front and back surfaces of the lens, are given, the coordinates of the remaining points can be determined in turn through numerical iteration. Then, we connect these points smoothly and in an orderly manner to obtain the contours of the front and back surfaces of the *f*-*θ* lens. Finally, we rotate these contours around the optical axis to construct a rotationally symmetrical *f*-*θ* lens, which can be employed in a two-dimension laser scanning system. Typically, the *f*-*θ* lens designed for the polygonal mirror is not rotationally symmetrical. Thus, the contours in the meridian plane can be designed by using the proposed method and the contours in the sagittal plane can be obtained in the same way by modifying the design parameters.

For a one-dimension laser scanning system, the *f*-*θ* lens is a cylindrical lens whose contour is axisymmetrical with respect to the optical axis, and the lens can be constructed by stretching these contours along the *z*-axis.

The focal length *f* and angle of field 2*θ*_max_ are set first in designing the double freeform surface *f*-*θ* lens. When different object distances *L* and center thicknesses *d* are selected, different contours can be obtained by using the above iterative method. Even for the same object distance, countless center thicknesses can be chosen to construct the lens contour. Similarly, there are countless object distances to choose for a certain lens thickness. This shows that even if the optical transformation requirements, i.e., the focal length and angle of field are fixed, the lens contours are not unique. This provides a greater degree of freedom for designing the double freeform surface *f*-*θ* lens. However, for different lens contours, the incident angles on the front and back surfaces are different, which makes the reflection loss and refraction different too. As a result, the transmittance and aberrations such as the field curvature of the different *f*-*θ* lenses differs from each other. Therefore, it is necessary to comprehensively consider the various performance indicators of all the potential *f*-*θ* lenses to select the most suitable one from them.

## 3. Simulation Analysis Results

A He-Ne laser with a wavelength of 632.8 nm is chosen as the scanning beam. The lens is made of polymethyl methacrylate (PMMA) with a refraction index *n* = 1.489 at the laser wavelength. Actually, other materials, such as Topas COC, which has comparable optical properties to PMMA, but exhibits better thermal–optical stability, are also acceptable. In practical applications, the optical performance, thermal–optical stability, and processing cost of materials should be taken into consideration. PMMA is chosen here because of its processing convenience by computer numerical control machine tools in the subsequent validation experiment.

### 3.1. Design and Simulation Analysis of F-θ Lens with Angle of Field of 60°

#### 3.1.1. Lens Design

**Figure 4:**
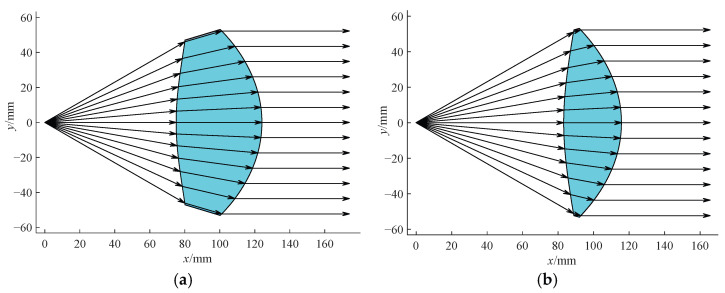
Optical transformation of the double freeform surface *f*-*θ* lens with a focal length of 100 mm and an angle of field of 60°. (**a**) Thick lens. (**b**) Thin lens.

When the rotating polygonal mirror is a regular 12-sided prism, the angle of field of the *f*-*θ* lens should be 360° × 2/12 = 60°. Nevertheless, the effective scanning angle should be smaller than 60°. The reason is that the laser beam has a certain width and must not exceed the mirror region. Considering the width of the scanning beam, the aperture angle of the designed lens should be slightly larger than the half-angle of field in order to avoid the influence of the lens and mirror edge on the beam transmission. Thus, the aperture angle *θ*_max_ = 30.5° ≈ 0.532 rad is taken. We set the focal length *f* = 100 mm, the object distance *L* = 75 mm, the center thickness *d* = 49 mm, and the angle discrete intervals, that is, scanning step size ∆*θ* = 1/8°. Using the numerical iterative method described in the second part, a lens contour is obtained as shown in Figure 4a. If the object distance is set as *L* = 83.2 mm and the center thickness is set as *d* = 32.5 mm, the designed lens of which the contour is shown in Figure 4b is thinner. The apertures of these two *f*-*θ* lenses are the same, which are 2*f* × *θ*_max_ ≈ 106.5 mm. These values are also their working ranges. Both lenses can transform the incident ray with a scanning angle of *θ* into an outgoing ray parallel to the optical axis with a height of *h = f* × *θ*.

#### 3.1.2. Simulation Analysis of Thin Lens

**Figure 5:**
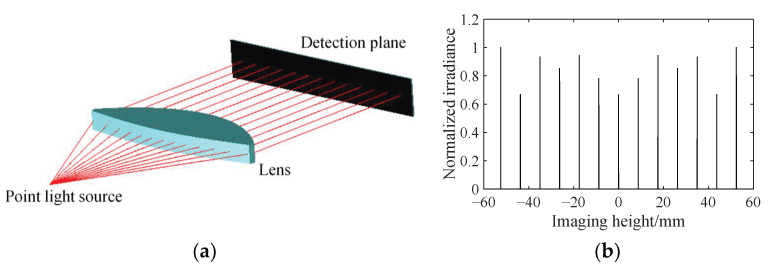
Simulation of the double freeform surface *f*-*θ* lens. (**a**) Ray tracing. (**b**) Imaging of scanning beams in the detection plane.

The thinner *f*-*θ* lens constructed by stretching the contours shown in Figure 4b along the *z*-axis is modeled in the optical software Zemax 2009. Obviously, it is a cylindrical lens and can be applied to a one-dimension laser scanning system. In general, the incident ray beam has a complicated wavefront when the polygonal mirror is adopted [25,34]. Here it is simplified as a thin beam just for addressing the validation of the proposed design method. In order to simulate the incident beams with different scanning angles, many Gaussian thin beams, of which the inclination angles increase from −30° to 30° at 5° intervals, are emitted from the origin. A detection is set at 100 mm away on the right of the object principal surface of the lens. Then Monte Carlo ray tracing is performed, as shown in Figure 5a.

Figure 5b shows the image of each beam in the detection plane after the transformation of designed lens. Based on this figure, the position of each spot, i.e., the image height, can be obtained. The power of each spot can also be calculated from this figure. Then the ratio of the power of each spot to the power of the incident beam is defined as the transmittance.

**Figure 6:**
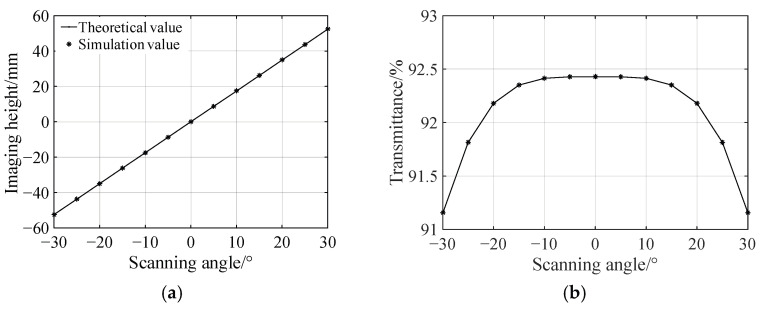
Simulation analysis of the designed *f*-*θ* lens. (**a**) Relationship between imaging height and scanning angle. (**b**) Relationship between transmittance and scanning angle.

The relationship between the image height and the beam scanning angle is demonstrated in Figure 6a, in which the asterisks indicate the simulation values and the solid line gives the theoretical values. It can be seen that the simulation values are in good agreement with the theoretical values. This fact demonstrates that the optical transformation of the designed *f*-*θ* lens possesses a very high linearity. The relationship between the simulated transmittance and the scanning angle is given in Figure 6b. The transmittance is not sensitive to the change in the beam scanning angle. When the scanning angle increases in absolute value, the transmittance decreases slightly, but basically is maintained around 92% over the entire field of view. This is because that the optimized object distance *L* and center thickness *d* make the two direction changes of each light beam caused by the refractions at the front and back surfaces of the designed *f*-*θ* lens occur to the same extent. Neither beam deflection exceeds 15°. According to the Fresnel formula, the Fresnel reflection losses of the light beams on the front and back surfaces are both about 4%. Thus, the designed *f*-*θ* lens possesses a high transmittance over the entire field of view, which will be of benefit to the imaging quality of the laser scanning system.

**Figure 7:**
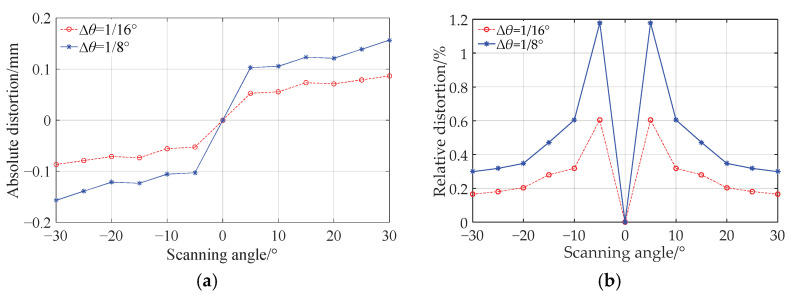
The error of the optical transformation. (**a**) Absolute distortion varies with scanning angle. (**b**) Relative distortion varies with scanning angle.

The error from the optical transformation of the *f*-*θ* lens is investigated precisely as follows. The difference between the simulation value and the theoretical value of the image height is defined as the absolute distortion, which varies with the scanning angle as shown by the blue asterisks and solid curve in Figure 7a. The odd symmetry of this curve is due to the symmetry used in the lens design. At the same time, this curve shows that the absolute distortion is basically in positive correlation with the scanning angle. The reason for this is that in the numerical iterative design of the lens, the angle discrete intervals are equal to each other. Thus, the distance between any two adjacent discrete points on both the front and back surfaces of the lens will inevitably increase when the scanning angle increases. When a double freeform surface *f*-*θ* lens is constructed using these discrete points, the sections between those widely spaced discrete points have less control of the rays. This fact will result in a bigger absolute distortion for bigger scanning angle. The relative distortion, which is defined as the ratio of the absolute distortion to the theoretical imaging height corresponding to a certain scanning angle, fluctuates slightly over the entire field of view with a maximum value of about 1.2%, as shown by the blue asterisks and the solid curve in Figure 7b.

Now we reduce the angle discrete intervals by half in the numerical iterative design of the lens, i.e., ∆*θ* = 1/16° is taken, and the other parameters remain unchanged. Then the *f*-*θ* lens is redesigned. The simulation values of the absolute and relative distortions of the new *f*-*θ* lens vary with the scanning angle as shown by the red circles and dashed curves in Figure 7a,b, respectively. The variation trend of distortion is similar to that of the *f*-*θ* lens designed with the larger discrete intervals ∆*θ* = 1/8°, but the variation range is reduced by about half. It can be seen that for an *f*-*θ* lens designed by the numerical iterative method proposed in this paper, its error value of the scanning linearity is determined by the angle discrete intervals.

#### 3.1.3. Simulation Analysis of Thick Lens

**Figure 8:**
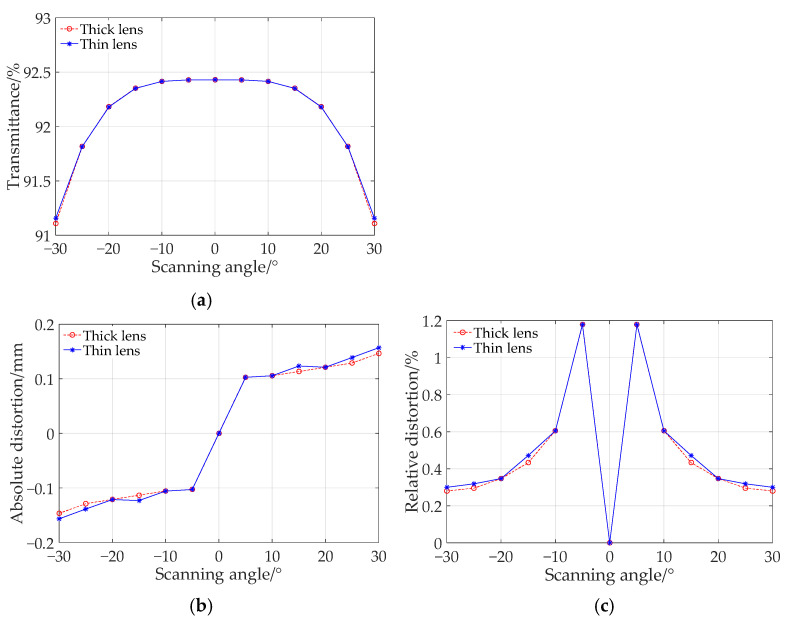
Comparison of scanning error of thick lens and thin lens. (**a**) Relationship between transmittance and scanning angle. (**b**) Absolute distortion varies with scanning angle. (**c**) Relative distortion varies with scanning angle.

For the thick lens given in Figure 4a, the relationship between the transmittance, absolute distortion, relative distortion, and the scanning angle is shown in Figure 8. As can be seen from the figure, the differences in optical performance between thick lens and thin lens are indeed quite small. Due to the influence of the materials, the thinner lens has less absorption, a weaker thermal effect, and smaller field curvature in practical applications.

A new double freeform surface *f*-*θ* lens with a focal length of *f*′ can be obtained by simply magnifying the coordinates of the above *f*-*θ* lens with a focal length of *f* = 100 mm by a factor of *f*′/*f*. Its characteristics of linearity and transmittance remain the same, but its working range will change to be 2*f*′ × *θ*_max_.

### 3.2. Design and Simulation Analysis of F-θ Lens with Angle of Field of 90°

**Figure 9:**
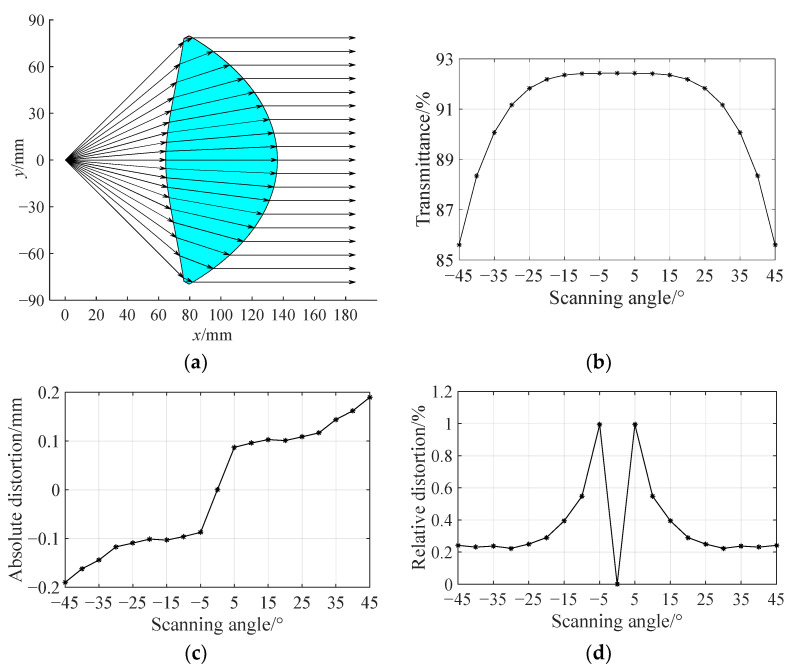
Double freeform surface *f*-*θ* lens with a focal length of 100 mm and an angle of field of 90°. (**a**) Optical transformation. (**b**) Transmittance varies with scanning angle. (**c**) Absolute distortion varies with scanning angle. (**d**) Relative distortion varies with scanning angle.

If the rotating polygonal mirror is a regular eight-sided prism, the angle of field of the corresponding *f*-*θ* lens should be 90°. The aperture angle of the lens is taken as *θ*_max_ = 45.5° in the lens design. Limited by computer hardware conditions, the angle discrete interval is still set as ∆*θ* = 1/8°. After setting the focal length *f* = 100 mm, the object distance *L* = 64.6 mm, and the lens thickness *d* = 71.6 mm, the lens contour is generated and shown in Figure 9a.

Figure 9b–d illustrate the transmittance, the absolute distortion and the relative distortion of the *f*-*θ* lens with an angle of field of 90° as a function of the scanning angle, respectively. Compared with those of the *f*-*θ* lens with an angle of field of 60° in Figure 6b and Figure 7a,b, it can be seen that for the *f*-*θ* lens with a different angle of field, the variation trends of the transmittance and the absolute distortion with respect to the angle of field are generally similar. Regarding the transmittance, when the absolute value of scanning angle exceeds 30°, the transmittance decreases significantly when the scanning angle increases. Nevertheless, the transmittance remains above 85% over the entire angle of field. Regarding the absolute distortion, when the scanning angle is small, the absolute distortion of the *f*-*θ* lens with an angle of field of 90° is slightly less than that of the *f*-*θ* lens with an angle of field of 60°. This is because, for the same focal length, the former lens has a smaller object distance, which results in smaller distances between discrete points and improves the ray control ability of the lens. For the *f*-*θ* lens with an angle of field of 90°, when the absolute value of scanning angle is less than 30°, its transmittance is identical to that of the *f*-*θ* lens with an angle of field of 60°.

## 4. Discussion

A numerical iterative design method of the *f*-*θ* lens in laser scanning systems is presented in this paper. In the point-by-point surface calculation process, the key idea is to calculate surface points and normal vectors using the Snell law and tangent continuity constraints. Different from the optimization design method of the traditional *f*-*θ* lens group or other point-by-point design methodologies of the freeform surface *f*-*θ* lens, this numerical iterative method can be used to obtain the two freeform surface contours of an *f*-*θ* lens simultaneously with less computational resources and lower design complexity. In this numerical iterative method, only two points and four vectors, all of which are two-dimensional, need to be calculated during each iteration. And the number of iterations is determined by the angle of field and the angle discrete interval.

According to the formula of the working range of the *f*-*θ* lens, if the given *f* and *θ*_max_ are bigger, the working range of the designed *f*-*θ* lens is bigger. But comparing these two double freeform surface *f*-*θ* lenses as illustrated in Figure 4b and Figure 9a, it can be seen that the thickness of the double freeform surface *f*-*θ* lens increases significantly as the angle of field increases. This will inevitably lead to an intensification of the field curvature. The uniformity of spot sizes and shapes in the detection plane corresponding to different scanning angles will degenerate as well. However, preliminary studies have found that Fresnel structures can be introduced into the front and back surfaces of the lens (especially the back surface) to reduce its thickness, so as to improve its comprehensive performance without affecting its optical transformation characteristic. Due to space limitations, the *f*-*θ* lens with Fresnel structure will not be discussed in this paper.

Linearity error, i.e., the relative distortion, is an important performance index for all the applications of a laser scanning system. According to the usage requirements, the linearity error of the *f*-*θ* lens needs to be controlled below 0.5%. The aforementioned simulation analysis shows that for an *f*-*θ* lens designed by the numerical iterative method proposed in this paper, its linearity error is determined by the angle discrete interval adopted in the designing process. Figure 7b illustrates that the *f*-*θ* lens designed with the angle discrete interval ∆*θ* = 1/16° has a linearity error below 0.5% except near the scanning angles +5°and −5°. In fact, it is not reasonable to specify a same linearity error for these scanning point near the center of scanning area based on the definition of the relative distortion (the spot position error divided by the required image height) [1]. If a lower linearity error is needed, the angle discrete interval should be reduced proportionally in the *f*-*θ* lens design process.

Further, the freeform surface contours obtained by this proposed method can be taken as the starting data for optimization in optical design software to achieve good image quality and scanning linearity.

## 5. Conclusions

In this paper, based on the ray mapping relationship of the *f*-*θ* lens and the construction theory of a double freeform surface lens, a numerical iterative design method of an *f*-*θ* lens is proposed. According to the specific design indexes, such as the angle of field and the focal length of the *f*-*θ* lens, and the appropriate iterative starting data, such as the object distance and the center thickness of the *f*-*θ* lens, the coordinates of each discrete point on the front and back surfaces of the *f*-*θ* lens can be solved successively by employing this method. And then the contour of the *f*-*θ* lens can be obtained.

The front and back surfaces of the designed *f*-*θ* lens are both freeform surfaces and thus have a strong control ability of rays. This *f*-*θ* lens has a small distortion over the entire field of view. And the variation range of distortion can be controlled by the angle’s discrete intervals adopted in the iterative computation. At the same time, the transmittance of the *f*-*θ* lens changes slightly over the entire field of view and remains at a high level. This will contribute to high-quality imaging or scanning.

In summary, the numerical iterative design method of the *f*-*θ* lens proposed in this paper is simple and efficient, consumes less computational resources, and can design a double freeform surface *f*-*θ* lens with a simple structure, compact size, large working range, controllable linearity, and high transmittance. This method provides a new idea for the design of *f*-*θ* lenses, which is of great significance for further improving the performance of laser scanning optical systems.

## Data Availability

Data underlying the results presented in this paper are not publicly available at this time but may be obtained from the authors upon reasonable request.

## References

[1] Marshall G.F., Stutz G.E. (2012). Handbook of Optical and Laser Scanning.

[2] May K.H., Mohammadzadeh S., Keil A., von Freymann G., Friederich F. (2024). Coherent Off-Axis Terahertz Tomography with a Multi-Channel Array and f-theta Optics. Sensors.

[3] Mohammadzadeh S., Hussung R., Keil A., Leuchs S., Krebs C., Nüßler D., Seewig J., von Freymann G., Friederich F. (2023). Compact hand-guided 3D scanning terahertz sensor platforms with 3D-printed aspherical telecentric f-θ lens. Int. J. Microw. Wirel. Technol..

[4] Pernechele C., Consolaro L., Jones G.H., Brydon G., Da Deppo V. (2021). Telecentric F-theta fisheye lens for space applications. OSA Contin..

[5] Zhou Q., Tian Y., Wang J., Xu M. (2020). Design and implementation of a high-performance panoramic annular lens. Appl. Opt..

[6] Novák J., Mikš A. (2023). Method for algebraic calculation of initial design parameters of a two-element telecentric f-theta lens. Appl. Opt..

[7] Wang W., Schmidt K., Wapler M.C., Wallrabe U., Czarske J.W., Koukourakis N. (2023). Fully refractive telecentric f-theta microscope based on adaptive elements for 3D raster scanning of biological tissues. Opt. Express.

[8] Chen C., Pu Y., Shi W. (2023). Low-cost spectrometer design for ultra-high resolution spectral domain optical coherence tomography. Chin. Opt. Lett..

[9] Park S.-H., Kim S.-D., Lee D.-H. (2019). Design of Telecentric F-theta Lens for 532 nm Wavelength Laser. J. Korean Ophthalmic Opt. Soc..

[10] Hu X., Hua H. (2014). High-resolution optical see-through multi-focal-plane head-mounted display using freeform optics. Opt. Express.

[11] Yang T., Zhu J., Jin G. (2014). Design of freeform imaging systems with linear field-of-view using a construction and iteration process. Opt. Express.

[12] Ok G., Park K., Chun H.S., Chang H.-J., Lee N., Choi S.-W. (2015). High-performance sub-terahertz transmission imaging system for food inspection. Biomed. Opt. Express.

[13] Zhu Z., To S., Zhang S. (2015). Active control of residual tool marks for freeform optics functionalization by novel biaxial servo assisted fly cutting. Appl. Opt..

[14] Kumar S., Tong Z., Jiang X. (2022). Advances in the design and manufacturing of novel freeform optics. Int. J. Extrem. Manuf..

[15] Aguirre-Aguirre D., Villalobos-Mendoza B., Díaz-Uribe R., Campos-García M. (2020). Null-screen design for highly freeform surface testing. Opt. Express.

[16] Gonzalez-Utrera D., Villalobos-Mendoza B., Diaz-Uribe R., Aguirre-Aguirre D. (2024). Modeling, fabrication, and metrology of 3D printed Alvarez lenses prototypes. Opt. Express.

[17] Zhang Z., Liu Y., Li C., Ding Y., Yang C., Zhao Y., Xue C. (2024). Precision glass molding technology for XY polynomial freeform optical elements with simulations and experiments. Opt. Express.

[18] Chang X., Hu Y., Wang J., Liu X., Hao Q. (2025). Dynamic Interferometry for Freeform Surface Measurement Based on Machine Learning-Configured Deformable Mirror. Sensors.

[19] Wang K., Han Y., Li H., Luo Y. (2013). Overlapping-based optical freeform surface construction for extended lighting source. Opt. Express.

[20] Kang X.L., Yao H.B., Liu Q.L., Wang L., Zhang B., Chen K. (2019). Secondary optical design of diffused illumination of extended light source based on refractive index precompensation method. Laser Optoelectron. Prog..

[21] Gao Z., Yuan Q., Li X., Chen L., Ye J. (2017). Review of optical freeform surface representation technique and its application. Opt. Eng..

[22] Chen C., Hao Q., Liu L., Cao J., Zhang Y., Cheng Y. (2024). 10× continuous optical zoom imaging using Alvarez lenses actuated by dielectric elastomers. Opt. Express.

[23] Yang Y., Wang J., Li Y., Bai J. (2023). Design of a panoramic annular lens system with an ultra-wide angle via an annular Gaussian radial basis function freeform surface. Appl. Opt..

[24] Zhong Y., Tang Z., Gross H. (2020). Correction of 2D-telecentric scan systems with freeform surfaces. Opt. Express.

[25] Zhang T., Chen H., Luo W., Du H., Cheng D., Wang Y., Wu R., Wang Y., Kidger T.E. Optical design of a wide linear field-of-view, high resolution, and compact f-theta laser scanning system. Proceedings of the SPIE Optical Design and Testing XII.

[26] Park Y.-W., Qin Z., Lyu S.-K. (2021). Study on design and processing performance verification of a 600 dpi f-theta lens. J. Mech. Sci. Technol..

[27] Harris Z.B., Katletz S., Khani M.E., Virk A., Arbab M.H. (2020). Design and characterization of telecentric f-θ scanning lenses for broadband terahertz frequency systems. AIP Adv..

[28] Xu C., Ye D.J., Liu H.Z., Cheng Z.X., Lin H.Z., Song W.T. (2025). Optical design of a freeform f-θ lens for the 405  nm laser scanning unit. Appl. Opt..

[29] Zhu J., Yang T., Jin G. (2013). Design method of surface contour for a freeform lens with wide linear field-of-view. Opt. Express.

[30] Ghasemi S.H., Hantehzadeh M.R., Sabbaghzadeh J., Dorranian D., Vatani V., Babazadeh A., Hejaz K., Hemmati A., Lafouti M. (2012). Designing a plano-convex aspheric lens for fiber optics collimator. Opt. Lasers Eng..

[31] Syu Y.-S., Wu C.-Y., Lee Y.-C. (2019). Double-Sided Freeform Lens for Light Collimation of Light Emitting Diodes. Appl. Sci..

[32] Gong C., Xu H., Xu C., Liang J., Mu Y. (2020). Design and prototyping of highly-collimated long-distance optical systems with an LED light source. Appl. Opt..

[33] Liu C., Liu J., Xing Y., Ao X., Shen H., Yang C. (2025). An Iterative Deflectometry Method of Reconstruction of Separate Specular Surfaces. Sensors.

[34] Kim W.-B., Moon S.-D., Kim H.-S., Yoo J.-H., Kim H.-C. (2009). Optical Design and Manufacturing Technology for High Resolution Laser Scanning Unit. Int. J. Precis. Eng. Manuf..

